# Detection of a sympatric cryptic species mimicking *Aedes albopictus* (Diptera: Culicidae) in dengue and Chikungunya endemic forest villages of Tripura, India, posing a daunting challenge for vector research

**DOI:** 10.1038/s41598-025-96146-9

**Published:** 2025-04-24

**Authors:** Saurav Biswas, Jadab Rajkonwar, Sasmita Rani Jena, Phiroz Gogoi, Tulika Nirmolia, Sathishkumar Vinayagam, Gautam Hazarika, Ashwarya Kumari Sihag, Bhaskar Borah, Rocky Pebam, Harpreet Kaur, Kalpana Baruah, Kanwar Narain, Sarala K Subbarao, Dibya Ranjan Bhattacharyya, Biswajyoti Borkakoty, Ipsita Pal Bhowmick

**Affiliations:** 1 Regional Medical Research Center- Northeast Region (RMRC-NE)-ICMR, Dibrugarh, 786001 India; 2 Regional Office of Health and Family Welfare, Kolkata, 700106 India; 3 Indian Council of Medical Research (ICMR), Ramalingaswami Bhavan, Delhi, 110029 India; 4 National Center for Vector Borne Disease Control, DGHS, 110054 MOHFW New Delhi, India; 5 Formerly Regional Medical Research Center- Northeast Region (RMRC-NE)-ICMR, Dibrugarh, 786001 India; 6 Formerly National Institute of Malaria Research, ICMR, 110077 Delhi, India; 7 Centre for Post Graduate & Research Studies, Periyar University, Dharmapuri, 635205 India; 8 National Health Mission (NHM), Biswanath, Assam India; 9 Department of Space, North East Space Application Centre (NESAC), Government of India, Umiam, 793103 India; 10 Department of Health Research, Model Rural Health Research Unit (MRHRU), Tripura, Govt Agartala, India

**Keywords:** *Aedes*, *Aedes nr. albopictus*, Rubber garden, Dengue, Tripura, Evolution, Molecular biology

## Abstract

The *Aedes* (*Stegomyia*) *albopictus* (Skuse, 1895) (Diptera: Culicidae) is one of the major vectors for Dengue and Chikungunya. However, our study uncovered another mosquito species morphologically similar to *Ae. albopictus* but is genetically different. The male genitalia of this species possess minute differences in the IX tergum with *Ae. albopictus*. Nucleotide diversity and mean genetic distance analysis confirmed the genetic difference from *Ae. albopictus* and other *Aedes* species. However, this species has a significant degree of genetic similarity with the cryptic species of *Ae. albopictus* earlier reported from Vietnam and China. The time tree revealed the median divergence time of this species and *Ae. albopictus* species to be approximately 36.13 million years ago. This study marks the discovery of an *Aedes nr. Albopictus* species resembling *Ae. albopictus* in India and third in the world, also reports the distinct morphological feature of the male genitalia for the first time. Our study indicates the sympatric behavior of this species as it shares the breeding habitat of *Ae. albopictus*. The absence of endosymbiont *Wolbachia* in this species raises the possibility of reproductive isolation with *Ae. albopictus* leading to sympatric speciation and increasing virus-carrying capability for this species, having significant implications for vector-borne disease control.

**Supplementary Information:**

The online version contains supplementary material available at 10.1038/s41598-025-96146-9.

## Introduction

Currently, within the medically significant *Stegomyia* subgenus of the genus *Aedes *there are 132 species recognized globally^1^. Among these, *Aedes* (*Stegomyia*) *aegypti* (Linnaeus, 1762) and *Aedes* (*Stegomyia*) *albopictus* (Skuse, 1895) are particularly well-known for being primary vectors of Dengue, Chikungunya, and Zika virus. However, other *Aedes* species within the *Stegomyia *subgenus, found in Africa and Asia, are also considered potential vectors for these viruses^2^.

Huang in 1979 included 37 species under the subgenus *Stegomyia *from the Oriental region and, categorized them into 5 species groups^3^. Of these, *Ae. albopictus *belongs to the Albopictus Subgroup under Scutellaris Group. The Scutellaris Group contains 8 species in the Albopictus Subgroup and 7 species in the Scutellaris Subgroup, all in the Oriental region^3^.

Identification within subgenus *Stegomyia *mostly relies on morphological characteristics, particularly patterns on the thorax and tarsi in adult mosquitoes. The Scutellaris Group, for instance, is marked by a distinct white stripe on the scutum. However, identifying adult females is challenging due to subtle differences in the morphological characters^4^. Huang (1975)^5^ also noted that larvae of many species in this group share similar breeding habitats, which can lead to misidentification, as was the case with *Ae. krombeini* being mistaken for *Ae. albopictus* in Sri Lanka. In India, *Ae. albopictus*, *Ae. novalbopictus*,* Ae. patriciae*,* Ae. pseudoalbopictus*,* Ae. subalbopictus*,* Ae. unilineatus*,* Ae. krombeini*,* Ae. malayensis*, and *Ae. scutellaris *have been reported under Scutellaris Group^6^.

Recent studies have identified a novel cryptic species of *Ae. albopictus* that coexists with *Ae. albopictus *in the forested regions of Vietnam and China living in sympatry^7,8^. Although this newly discovered species is morphologically similar to *Ae. albopictus*, molecular analysis, and cibarial armature morphology revealed this species to be distinct. Cryptic species have also been reported in mosquito genera such as *Anopheles* and *Culex.* About 30 *Anopheles *vector species have been identified as species complexes and the number of cryptic/ isomorphic species identified so far varies in each complex (WHO, 2007)^9^.

*Ae. albopictus* commonly hosts *Wolbachia* endosymbiont, which may cause CI (cytoplasmic incompatibility) in mosquitoes, *Wolbachia *induced CI may give protection to mosquitoes against various RNA viruses^10^. This underscores the importance of accurate species identification and also detection of *Wolbachia* infection in mosquito species.

In our previous investigations in rural forested areas of Tripura, we identified molecularly confirmed *Ae. albopictus *species, whereas some specimens showed differences in the molecular studies^11^. In this current study, we have characterized these samples, along with other archived *Aedes* larva and adult samples from different areas in Tripura, collected between 2019 and 2022.

## Results

### Morphological identification

180 adult mosquitoes were identified as *Aedes* species. Out of 180, 139 resembled *Ae. albopictus,* and 15 were damaged *Aedes* species. So, these 15 damaged *Aedes *specimens and 139 that resembled *Ae. albopictus *were subjected for molecular identification. 

### ITS2 PCR results

**Fig. 1:**
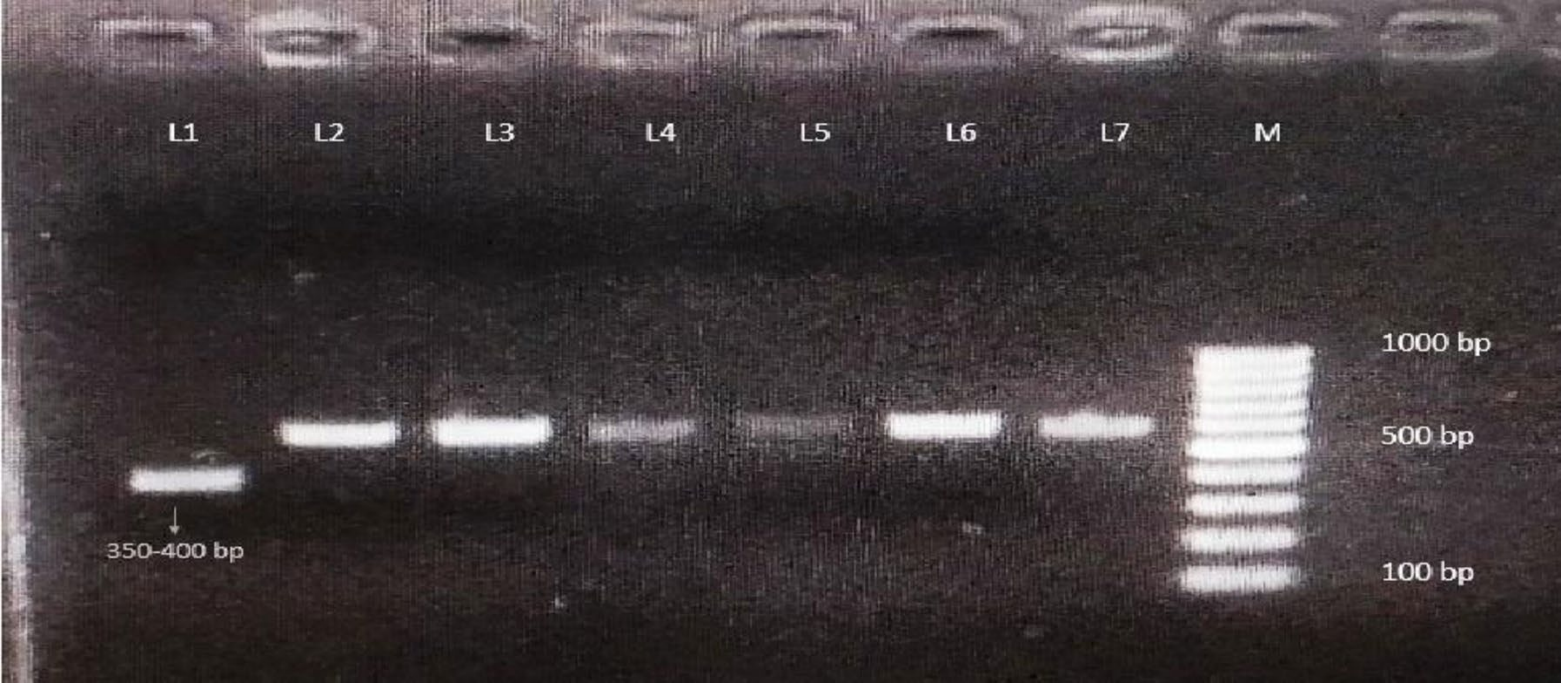
Agarose gel electrophoresis of ITS2 gene shows different *Aedes* species as collected from Tripura. L1 represents one species different from *Ae. albopictus *(band size between 350-400 bp). L2-L7 represents *Ae. albopictus *and M represents 100 bp DNA ladder.

In our study, 125 samples showed the typical band size of 550 bp. This particular band size has been previously identified as characteristic of the *Ae. albopictus*^11^. Fourteen samples showed band size between 350 and 400 bp and were subjected to sequencing analysis (Fig. 1).

### DNA sequence analysis

In this study, we analyzed 14 DNA samples that demonstrated a band size of 350–400 bp in the ITS2 PCR test. These specimens were subsequently sequenced for both the COI and ITS2 genes. Out of the 14 specimens, successful sequencing was achieved for 13 specimens by either COI or ITS2, (5 for COI and 9 for ITS2). One specimen was successfully sequenced for both genes.

**Fig. 2:**
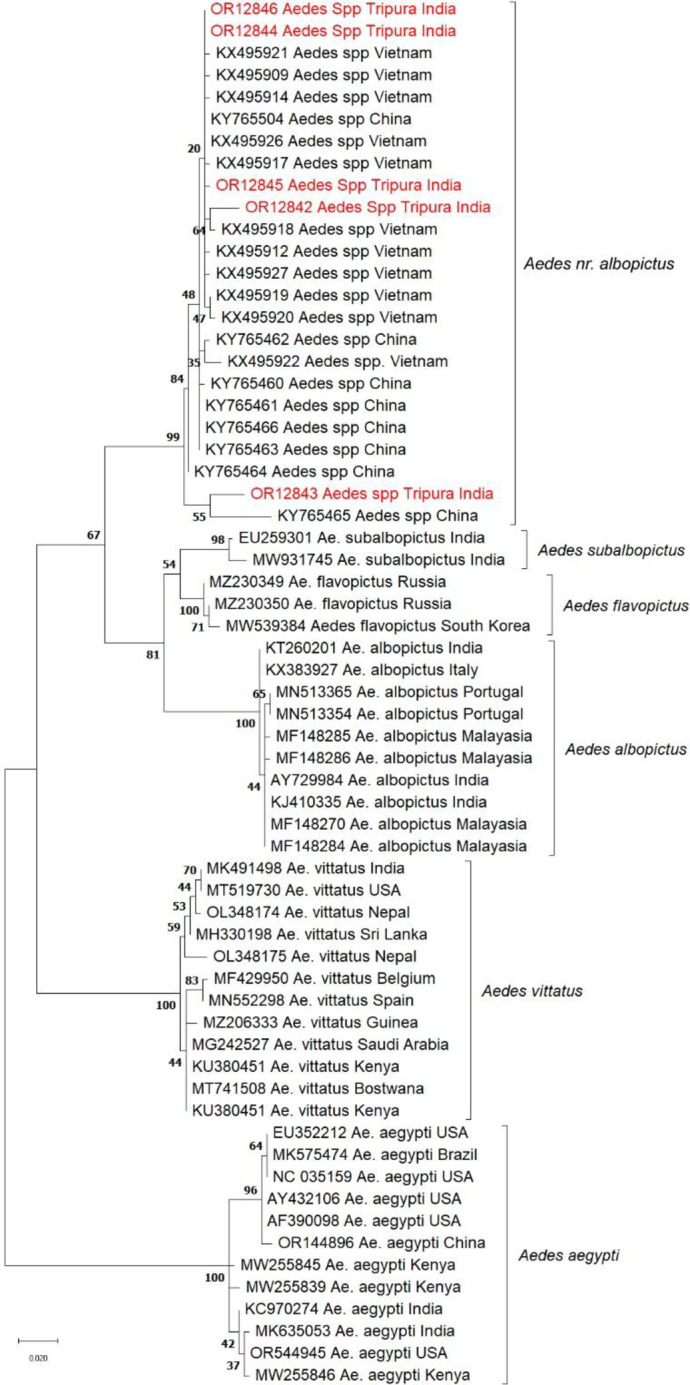
COI phylogenetic tree of *Aedes* mosquito, Tripura was inferred using the Maximum Likelihood method based on the best-fit model (General time reversible + Gamma) by using a bootstrap value of 500.

**Fig. 3:**
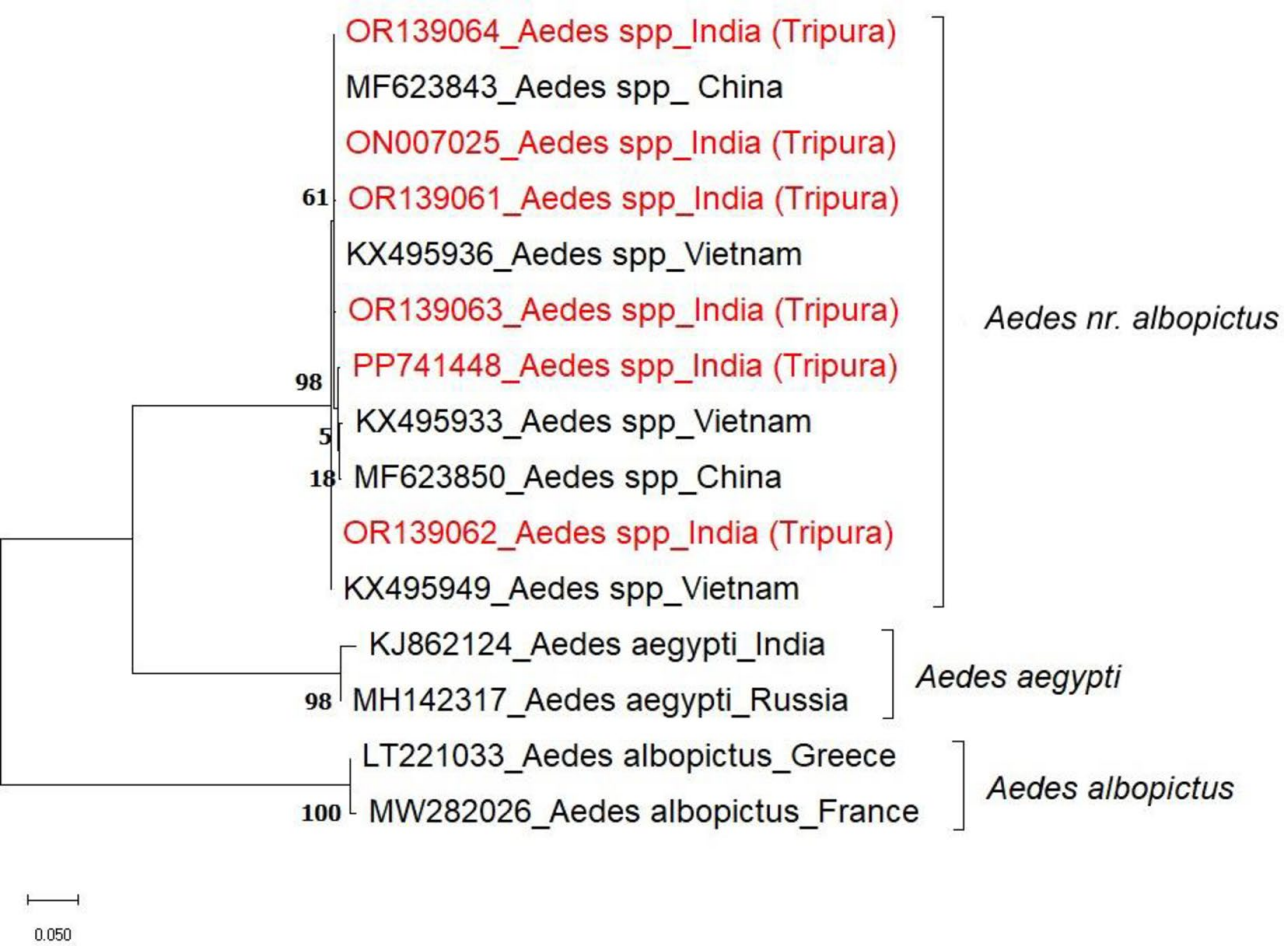
ITS2 phylogenetic tree of* Aedes *mosquito, Tripura inferred by using the Maximum Likelihood method based on the best fit model (Kimura 2-parameter) by applying a bootstrap value of 500.

The blast similarity search for the 5-COI and 6-ITS2 sequences (Accession numbers: OR139061-OR139064, ON007025, PF741448, OR129842-OR129846) revealed to be similar to *Ae. albopictus *cryptic species earlier reported from Vietnam and China^7,8^ (Figs. 2 and 3). The remaining three ITS2 sequences did not match any sequence existing in the GenBank. Two specimens were found morphologically similar to *Ae. annandalei*, and one was morphologically close to the genus *Topomiya* (RMRC unpublished data).

### Phylogenetic analysis

The phylogenetic analysis of these COI sequences was performed alongside those previously reported *Ae. albopictus *cryptic species from Vietnam and China^7,8^.

Additionally, sequences of *Ae. albopictus* from India and other global regions, *Ae. subalbopictus* from India, *Ae. vittatus* and *Ae. aegypti* were included (Fig. 2).

Similarly, in the ITS2-based phylogenetic tree, our sequences were in the same clade along with the *Ae. albopictus *cryptic species from Vietnam and China^7,8^, along with *Ae. albopictus*, and *Ae. aegypti* as outgroup. Notably, in both phylogenetic trees, the isolates from this study clustered closely with those from Vietnam and China (Figs. 2 and 3). We have provisionally designated *Aedes albopictus * cryptic species found in our study as *Aedes nr. albopictus*.

### Nucleotide diversity and mean genetic distance analysis

Very low nucleotide diversity and mean genetic distance of this study population was recorded with the population of *Ae. albopictus* cryptic species of China and Vietnam respectively. However, the nucleotide diversity and mean genetic distance with *Ae. albopictus* and other *Aedes* were comparatively higher (Tables 1 and 2).

**Table 1:** Nucleotide diversity (Pi) and nucleotide difference (K) between *Ae. nr. albopictus* with *Ae. albopictus* cryptic species Vietnam, China and other *Aedes* species.

*Ae. nr. albopictus*	*Ae. albopictus* cryptic species Vietnam	*Ae*. *albopictus *cryptic species China	*Ae. flavopictus*	*Ae. subalbopictus*	*Ae. albopictus*	*Ae. vittatus*	*Ae. aegypti*
Nucleotide Diversity (Pi)	0.00410	0.00273	0.05328	0.05328	0.0667	0.07423	0.08880
Nucleotide Difference (K)	1.500	1.00	19.500	19.500	21.833	27.167	32.500

**Table 2:** Mean genetic distance between the *Aedes* species.

	*Ae. nr.* *albopictus*	*Ae. albopictus* cryptic species China	*Ae. albopictus* cryptic species Vietnam	*Ae.* *subalbopictus*	*Ae.* flavopictus	*Ae.* *albopictus*	*Ae.* *vittatus*	*Ae.* *aegypti*
*Ae. nr. albopictus*	0.0000							
*Ae. albopictus cryptic China*	0.0307	0.0000						
*Ae. albopictus cryptic Vietnam*	0.0216	0.0209	0.0000					
*Ae. subalbopictus*	0.0895	0.0848	0.0823	0.0000				
*Ae. flavopictus*	0.0900	0.0849	0.0818	0.0402	0.0000			
*Ae. albopictus*	0.1046	0.1035	0.0951	0.0678	0.0540	0.0000		
*Ae. vittatus*	0.1192	0.1159	0.1189	0.1047	0.1228	0.1368	0.0000	
*Ae. aegypti*	0.1595	0.1571	0.1560	0.1526	0.1425	0.1372	0.1617	0.0000

### Time tree analysis

Time tree analysis gave an approximate date of divergence between the *Aedes* species included in this study. The median divergence time of *Ae. albopictus* and *Ae. albopictus* cryptic species in the analysis was found to be 36.13 million years ago, *Ae. subalbopictus* diverged from *Ae. albopictus* around 21.75 million years ago whereas *Ae. flavopictus* diverged from *Ae. subalbopictus* around 18.11 million years ago. From the analysis, it was revealed that *Ae. aegypti* (46.01 million years ago) was the most ancient ancestor among the species included in the time tree estimation. (Fig. 5).

### Haplotype analysis

Focusing on the five isolates of *Ae. nr. albopictus* from our current study, we identified four unique haplotypes. These are designated as Hap_1, Hap_2, Hap_3, and Hap_4, each representing a new haplotype discovered from this study. Meanwhile, Hap_3 aligned with *Ae. albopictus* cryptic sequences previously reported from China and Vietnam, indicating a shared haplotype with these regions. This analysis contributes to the growing understanding of the genetic diversity within the *Ae. albopictus* species, particularly highlighting the newly identified haplotypes in the Indian context. It also underscores the importance of continuous monitoring and genetic characterization of mosquito populations and understanding their distribution and evolution.

### Dissection of male terminalia

In our study, the morphology of adult male and female specimens closely resembled those of *Ae. albopictus*, as evidenced by the characteristic median white stripe on the scutum^4,13^ [Figure 6 (A), (B)]. A detailed examination of the male terminalia in three male specimens revealed that two were consistent with *Ae. albopictus*, identified by the horn-like median projection (MP) on their IX tergum [Figure 6 (C), (D)]. However, the IX tergum of the third specimen [Figure 6 (C)] presented slight variations, though it still resembled to *Ae. albopictus*, rather than any other species in the Scutellaris Group as described by Huang in 1972 for the oriental region^4^. Specifically, the middle projection of the IX tergum in this third specimen was observed to be narrower and smaller compared to the broader and larger structure typically seen in *Ae. albopictus* [Figure 6 (D)]. Furthermore, the two side lobes of the IX tergum in this specimen exhibited several prominent hairs, differing from the pattern observed in *Ae. albopictus*. These morphological observations highlight subtle yet significant variations within the specimens, contributing to our understanding of the diversity and morphological range within the *Ae. albopictus* species, particularly in the context of the Scutellaris Group in the oriental region. Specific confirmation of these three male specimens were also carried out by molecular methods.

**Fig. 4:**
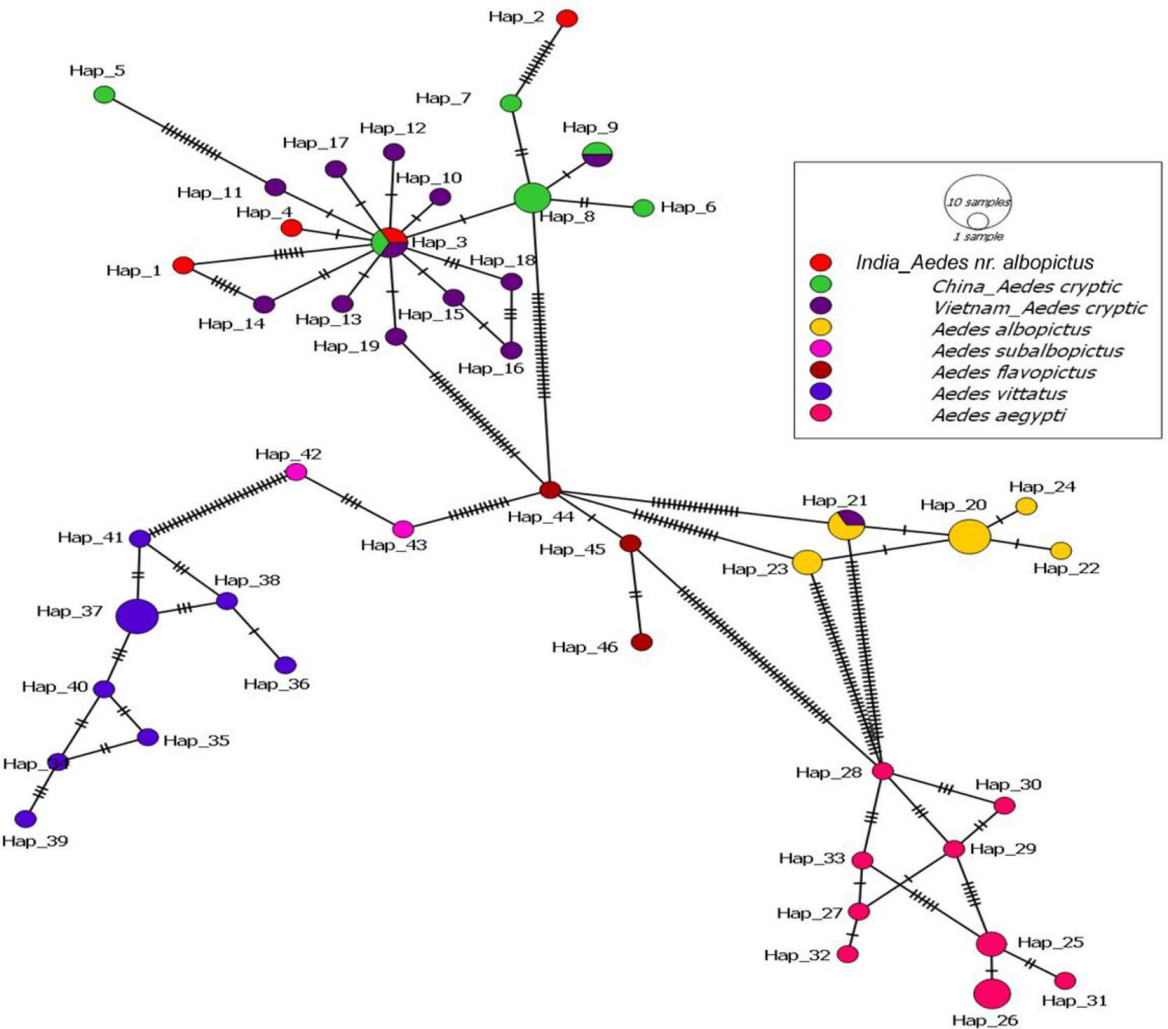
A minimum spanning network was used to generate a haplotype network of *Ae. albopictus*, *Ae. albopictus* cryptic species, *Ae. nr. albopictus*, *Ae. subalbopictus*, and other *Aedes *species. The haplotype of the current investigation is highlighted in red.

**Fig. 5:**
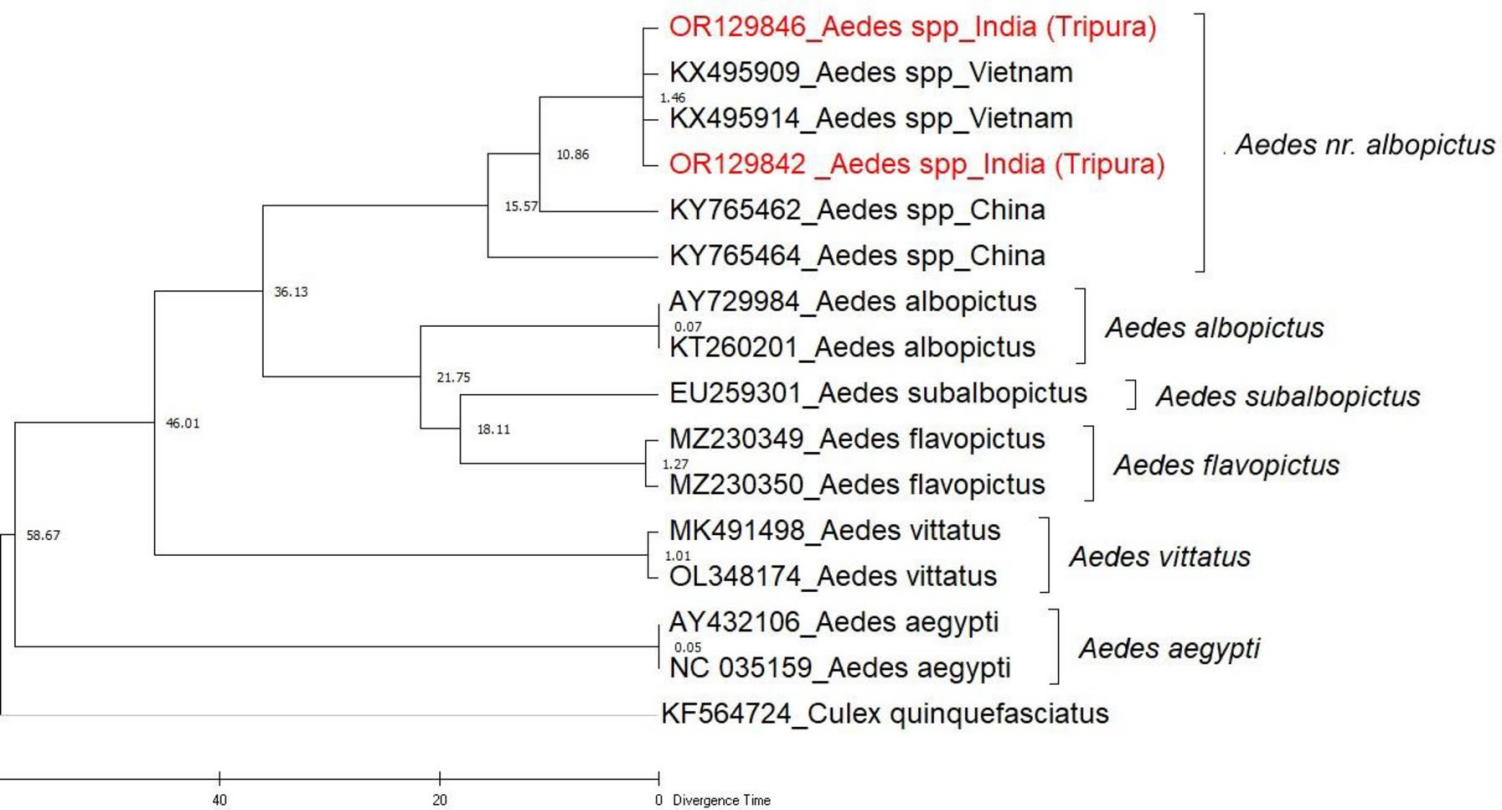
Time tree of *Aedes *species inferred by applying the RelTime method in MEGA X.

### Detection of *Wolbachia*

*Wolbachia* was found to be present naturally in the 7 *Ae. albopictus* specimens out of 9. However, it was absent in all the 10 *Ae. nr. albopictus* via amplification of the wsp gene (Supplementary Figure).

## Discussion

Reproductively isolated isomorphic and sympatric populations under a taxon are called cryptic or sibling species (WHO, 1998)^12^. Cryptic species are common in insects. It was estimated that for each morphologically identified insect species, there can be on average 3.1 cryptic species (Ni & Weins, 2022)^13^.

Adults of the Scutellaris Group of mosquitoes are morphologically characterized by having a median longitudinal white stripe of narrow scales from the anterior margin of the scutum to the wing root. However, they are most difficult to differentiate because of subtle differences and variation in their morphological characters (Huang, 1972)^4^. The presence of isomorphic species like *Ae. subalbopictus* and *Ae. pseudoalbopictus* along with *Ae. albopictus *creates a significant challenge in correctly identifying such species^7–9,11^. In addition, the presence of a cryptic species clearly emphasized the need for a molecular-based species identification in such a situation. In our present study, out of 139 morphologically resembled *Ae. albopictus* specimens,14 were found to be other species when subjected to molecular methods.

The phylogenetic trees, nucleotide diversity & mean genetic distance analysis confirmed that our study population is genetically different from *Ae. albopictus*, *Ae. subalbopictus* and other *Aedes* species. Although, this species is in the same clade as the *Ae. albopictus* cryptic species earlier reported from Vietnam and China (Tables 1 and 2), we have discovered three new haplotypes (Hap_1, Hap_2, and Hap_4), (Fig. 4). Also, this is the first time we have shown the sympatrically co-breeding of *Ae. nr. albopictus* with *Ae. albopictus* in rubber collection bowls.

The time tree analysis revealed the median divergence time of the *Ae. nr. albopictus* species and *Ae. albopictus* species to be approximately 36.13 million years ago and *Aedes aegypti* was the most ancient ancestor (approximately 46.01 million years ago). The divergent time between *Ae. albopictus* and *Ae. aegypti* found in our study is similar to those studies reported earlier by Zhao et al.^14^ and Nardi et al.^15^. These findings support the speciation of *Ae. nr. albopictus* species during the course of evolution.

**Fig. 6:**
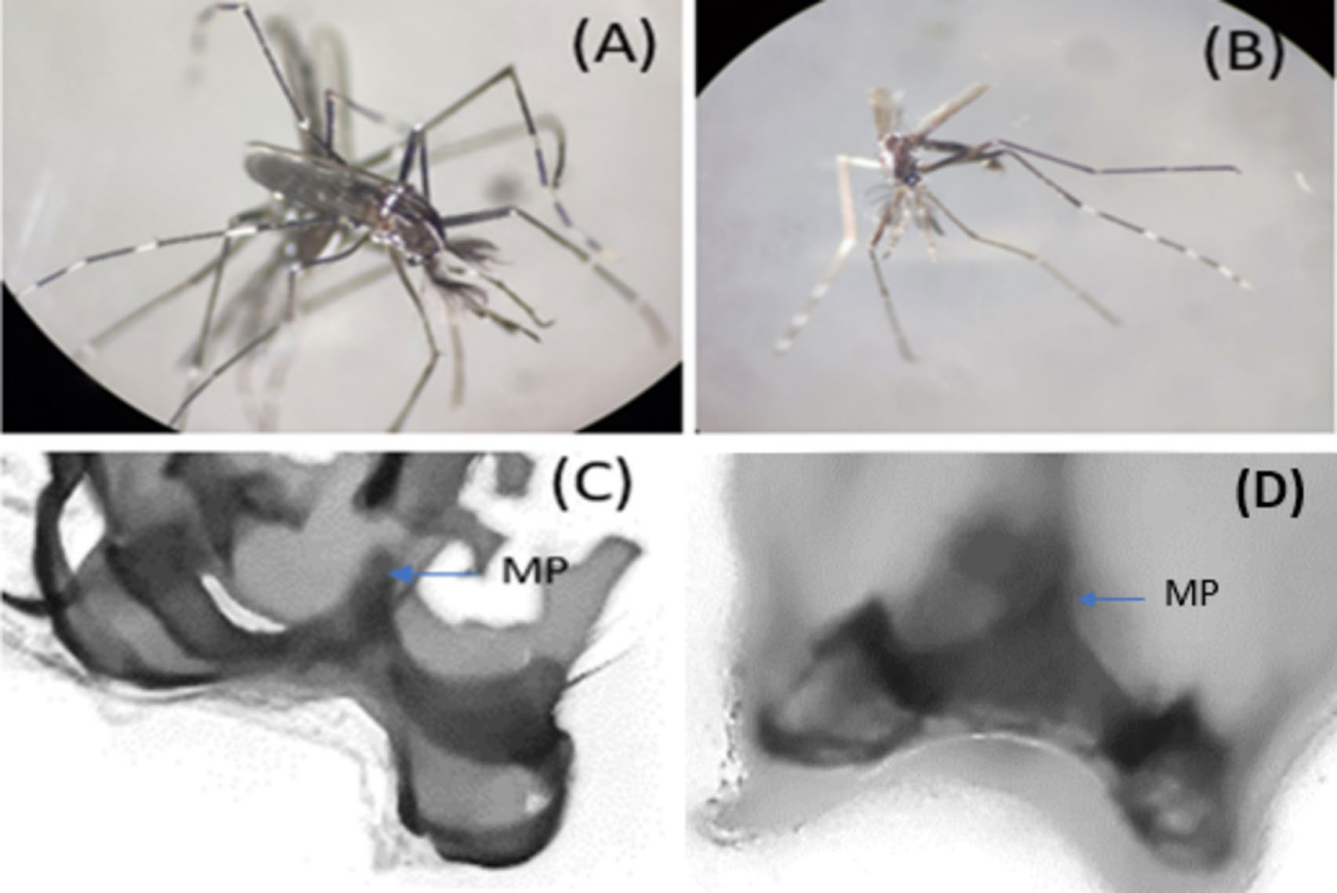
(**A**) *Ae. nr. Albopictus* and (**B**) represents *Ae. albopictus*. (**C**) and (**D**) represent the dissected images of IX tergum of male *Ae. nr. Albopictus* and *Ae. albopictus *respectively with conspicuous horn-like median projection (MP) in tergum IX.

In our present study the *Ae. nr. albopictus* specimens were morphologically very similar to those of *Ae. albopictus* (Fig. 6A). It was reported earlier that adult females of Scutellaris Group are most difficult to identify because of minute differences and variation in their taxonomic characters (Huang, 1972)^4^. However, the male genitalia of such species contains distinct characters (Huang,1972)^4^. Considering the same we have tried to examine male specimens of *Ae. nr. albopictus* species. Even though we could examine only one male specimen, we have observed subtle differences in the IX tergum of male genitalia of the *Ae. nr. albopictus* with *Ae. albopictus* [Figure 6 (A), (C)].

Although only one male specimen with differences in genitalia characteristics was found, it does suggest that this is a distinct species different from *Ae. albopictus* found in the area. Phylogenetic analyses (Figs. 2 and 3) conclusively indicate the presence of this species which is morphologically similar to *Ae. albopictus* but genetically different from *Ae. albopictus*, *Ae. subalbopictus*, * Ae. flavopictus*, *Ae. aegypti*,* Ae. vittatus*, while resembling *Ae. albopictus* cryptic species from China and Vietnam. Hence this is the first study to confirm the presence of a distinct *Aedes* species. In view of the evidence seen morphologically of male specimen, molecular distinctness between the study population and *Ae. albopictus*, we provisionally designate this species found in Dhalai, Tripura as *‘Aedes nr. albopictus’* following the nomenclature suggested by Sigovini et al.^16^, . Here nr. stands for near meaning is that species is close to *Ae. albopictus* but not identical. Further studies are required to formally describ this species as per the guidelines of International Code of Zoological Nomenclature (ICZN). This discovery marks for the first time such a species has been identified in this subcontinent region, contributing significantly to our understanding of the genetic diversity and distribution of the *Aedes* species in India.

The specimens of the *Ae nr. albopictus* in this study were from light trap collection in the huts, situated in the forested region (Fig. 6), thus are similar to collections in Vietnam^7^.

Several questions are still unanswered or only partially answered because *Ae. nr. albopictus* species was found for the first time in Tripura state, India. These include whether this species has breeding habitat preference, whether both pre- and post-mating barriers or only one of them responsible for the evolution of *Ae. nr*. *albopictus* species found in this study and what are the behavioural differences, like resting and feeding preferences, responses to existing vector control tools, vectorial potential as *Wollbachia* was not seen in this species, while it is present in *Ae. albopictus*. Moreover, what evolutionary processes in mosquito cryptic species lead to reproductive isolation in the absence of morphological distinction is yet to be deciphered, though some studies on cryptic species have tried to understand the aspect of reproductive isolation^17^. While emerging species of the malaria vector *Anopheles gambiaen* complex, *Anopheles culicifacies* complex have been shown to exhibit varied ecological preferences and strong pre and / or postzygotic reproductive isolation and cryptic species of *Ae. mariae* and *Ae. zammitii* under Mariae species complex have demonstrated post-mating reproductive isolation. However, there is still minimal or no data reporting reproductive isolation in *Culex* and *Aedes albopictus *mosquito cryptic species^17,18^.

Our study clearly indicates that these are sympatric species as both *Ae. albopictus* and *Ae. nr. albopictus* species were found in the same breeding habitat in the rubber collection bowls (Fig. 6). Sympatric speciation is a process whereby two closely related species occupying the same ecological niche undergo divergent evolution, leading to genetic differentiation that ultimately prevents them from interbreeding, and/or *Wolbachia* present in *Ae. albopictus *played a role in speciation. This sympatric association is an evolutionary mechanism that facilitates the emergence of new cryptic species^19^. Some studies have tried to answer whether *Wolbachia* infection has an impact on the reproductive isolation of mosquitoes. Researchers have identified *Wolbachia*-mediated reproductive isolation, particularly through unidirectional cytoplasmic incompatibility (CI), as a mechanism that could lead to population divergence and potentially contribute to the formation of new species^20^. *Wolbachia *infection can cause CI in insects such as mosquitoes, fruit flies, and butterflies. Crosses between infected males and uninfected females result in infertility. This one-way reproductive barrier could eventually lead to genetic divergence and reproductive isolation between populations, potentially driving speciation^20^. However, it’s important to note that reproductive isolation is often a complex process involving multiple mechanisms. While *Wolbachia*-mediated CI can be a significant factor, it may not be the sole driver of reproductive isolation between populations. Other factors, such as behavioural isolation, where individuals from different populations have preferences for mating with their own kind, and hybrid inviability, where hybrids between different populations have reduced fitness or viability, can also contribute to reproductive isolation^20^. In cases where crosses between infected males and uninfected females result in infertility due to *Wolbachia*-induced CI, the other direction of the cross (infected females with uninfected males) may remain fertile^20^.

Interestingly while *Ae. albopictus* is mostly harbouring *Wolbachia* in the guts, no *Wolbachia* was found in the *Ae. nr. albopictus *species in our studies or previous study^7,8^.

The lack of *Wolbachia* endosymbiont in our study, its near-absence in China’s *Aedes albopictus* cryptic species, suggests that *Wolbachia *may have played a role in its speciation^20^.

Moreover, the absence of a *Wolbachia *endosymbiont in this species also raises the possibility of increasing virus-carrying capability^21^ Viral detection could not be done because samples were used for studies to confirm the new species. Thus, this study calls for future studies that screen for RNA viruses like dengue, chikungunya, and rift valley fever to plan new vector control tools.

Our molecular approach, coupled with morphological studies, reaffirms the importance of such methods in identifying and confirming morphologically similar species, as highlighted in our previous studies^11^. Molecular identification not only aids in genetic characterization but also helps in accurately identifying breeding habitats shared by isomorphic species which would have otherwise been considered *Ae. albopictus*, only by morphological examination. The discovery of these species, particularly in the absence of molecular studies in India, suggests the possibility of missing more such isomorphic species in the past, underscoring the need for further research on archived samples.

## Conclusion

This study marks the discovery of *Ae. nr. albopictus* species resembling *Ae. albopictus* in India. Our findings from Tripura, India highlight the possibility of sympatric speciation of *Ae. nr. albopictus* species. This is a significant development in understanding the importance of molecular characterization, identification of breeding sites, evolution of *Aedes* species and transmission dynamics of Dengue and Chikungunya in rural, forested areas of Tripura, India.

## Materials and methods

### Study area and collection of mosquitoes

**Fig. 7:**
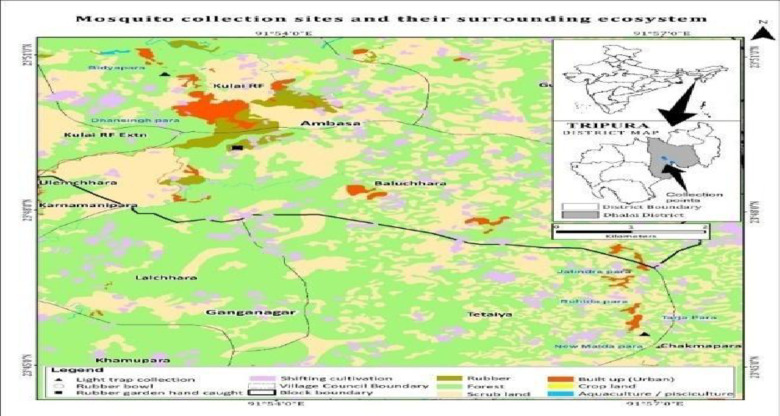
LULC map showing geolocation of the *Ae. nr. albopictus *and collection areas from Tripura.

This study was conducted using archived *Aedes* samples collected from five districts of Tripura: Dhalai, Gomoti, Sepahijala, Unokoti, and West District from 2019 to 2022. Mosquito larvae were collected from various natural and artificial containers and adult mosquitoes were caught in the traps or hand-caught (Fig. 7).

### Morphological identification of mosquitoes

For morphological identification, we followed the standard keys^4,22^. The adults emerging from larvae, along with adults caught in light traps and by hand (Mouth aspirator), were first identified based on their morphological features. Male genitalia of three adult males were examined by dissecting the male terminalia and by mounting them on glass slides following the book (Huang, 1972)^4^.

### Molecular identification of mosquito species

To perform molecular identification, genomic DNA was extracted from individual larvae and the legs of adult mosquitoes. Using a 1.5 mL centrifuge tube, the whole larva or mosquito’s leg was grinded by using a micro pestle and then QIAamp DNA micro kit’s DNA extraction methodology was followed to extract the total nucleic acids. The DNA was stored at −20ºC for further analysis. We targeted the mitochondrial cytochrome oxidase I (COI) and Internal Transcribed Spacer II (ITS2) regions. For COI region, briefly the PCR was performed in 50 µL reaction volume with approximately 50 ng DNA as a template using 2X master mix (Promega, USA) following methodologies described by Kumar et al.^23^. Additionally, MgCl_2_ (1.5 mM) was added in the final reaction as part of modification. For ITS2 region amplification a gradient PCR protocol was employed following the primer pairs described by Walton et al.^24^. 56 ºC was taken as annealing temperature since the annealing temperature described in the reference protocol did not yield positive result. The final PCR was carried out in a 50 µL 2X master mix (GoTaq Promega, USA) and 1.5 mM MgCl_2_ with approximately 50 ng DNA as a template. Post electrophoretic separation, PCR products were visualized under UV Transilluminator (BioRad XR).

### Sequencing of COI and ITS2 genes and phylogenetic analysis

Following post-PCR clean-up, the amplicons were bi-directionally sequenced at Eurofins Genomics, India. Quality value greater than or equal to Qv20 was considered for base calling. Sequence editing was performed in Bio-edit v7.0.5.3 software^25^and submitted to the GenBank. Similar sequences were retrieved from NCBI, and phylogenetic analysis was done in MEGA X software^26^. The phylogenetic trees for the COI and ITS2 genes were constructed by using the Maximum Likelihood method based on the best fit model^27^. For the COI tree construction 12 *Ae. aegypti*, 12 *Ae. vittatus*, 10 *Ae. albopictus*, 3 *Ae. flavopictus*, *2 Ae. subalbopictus*, 8 *Ae. albopictus* cryptic species of China, 11 *Ae. albopictus* cryptic species of Vietnam and 5 of this study were used. In the ITS2 phylogenetic tree 2 *Ae. albopictus*, 2 *Ae. aegypti*, *Ae. albopictus* cryptic species of China, 3 *Ae. albopictus* cryptic species of Vietnam and 6 of this study were used. The length of the alignment used to construct the phylogenetic tree for COI and ITS2 gene were 396 and 331 base pairs respectively.

### Nucleotide difference and diversity analysis

The nucleotide difference and diversity among the different *Aedes *species were calculated for the COI gene using the software DnaSP v.6^28^.

### Mean genetic distance analysis

The mean genetic distance among the different *Aedes *species was calculated for the COI gene using the MEGA X software^26^.

### Time tree analysis

For the estimation of the time tree, the approximate divergence time between *Ae. aegypti* and *Ae. albopictus *was calculated using the time tree of life online database^29^with a class interval of 15–59 million years ago^14,15,30^. The time tree was constructed in MEGA X software^26^ using the mitochondrial COI sequences of *Ae. albopictus* cryptic (India, China and Vietnam). *Ae. albopictus*, *Ae. subalbopictus*, *Ae. vittatus* and *Ae. aegypti*. The COI sequence of *Culex quinquefasciatus *was included as an outgroup. The time tree was inferred by applying the ReLTime method to the constructed phylogenetic tree^27^. Confidence intervals and the minimum and maximum time boundaries on nodes for which calibration densities were provided, were computed using the Tao et al.^31^ method. The length of the alignment for inferring the time tree was 367 base pairs.

### Haplotype network analysis

For haplotype network analysis, we used PopART v.1.7 software^32^ and the minimum spanning method for analyzing variation in the mitochondrial COI region. This included sequences from both *Ae. albopictus* cryptic species sampled in Vietnam and China, as well as other *Aedes* taxa collected from diverse geographic locations around the globe. A total of 62 COI sequences, including the five detected in our study, were incorporated into the haplotype network analysis.

### Detection of *Wolbachia* in mosquito species

To detect *Wolbachia* endosymbiont in mosquitoes, we extracted genomic DNA from the abdomen of adult mosquitoes and individual larvae following the DNA extraction protocol of the QIAamp DNA mini kit. The presence of *Wolbachia* was indicated by a 600 bp amplicon in the PCR analysis. PCR was carried out by targeting the *Wolbachia* surface protein (wsp) gene using the primer pair wsp81F/wsp691R as previously described by Braig et al.^33^. The PCR reaction was performed in 50 µL reaction volume using 2X master mix (Promega, USA) with approximately 50 ng of gDNA as template and at an annealing temperature of 55ºC. Known *Wolbachia* DNA was taken as positive control whereas DNA extracted from the leg of *Aedes* mosquito was taken as negative control. PCR products were analyzed in ethidium bromide-stained 1.5% agarose gel and viewed under UV Transilluminator (BioRad XR).

### Preparation of ecological map with cases and vectors

Utilizing ortho-rectified Indian Remote Sensing satellite data, Cartosat-1 (2.5 m) and LISS-IV (5.8 m), land use and land cover (LULC) mapping were produced utilizing on-screen visual interpretation techniques on the Geographic Information System (GIS) platform. Using the most recent spatial layer data (2019), which was first supplied at 1:10,000 scales by NRSC/ISRO’s Space-based Information Support, major LULC categories and subcategories were delineated and updated. The project team also carried out field verifications to guarantee the accuracy of the data interpretations.

## Electronic supplementary material

Below is the link to the electronic supplementary material.

Supplementary Material 1

## Data Availability

Sequences dataset that supports this study (newly discovered cryptic species of *Aedes albopictus*/ *Ae. nr. albopictus*), have been submitted to the GenBank database ( NCBI) — Accession Numbers OR139061-OR139064, ON007025, PF741448 and Accession Numbers OR129842-OR129846 respectively.
