# Correction: Detection of a sympatric cryptic species mimicking *Aedes albopictus* (Diptera: Culicidae) in dengue and Chikungunya endemic forest villages of Tripura, India, posing a daunting challenge for vector research

**DOI:** 10.1038/s41598-025-32139-y

**Published:** 2025-12-18

**Authors:** Saurav Biswas, Jadab Rajkonwar, Sasmita Rani Jena, Phiroz Gogoi, Tulika Nirmolia, Sathishkumar Vinayagam, Gautam Hazarika, Ashwarya Kumari Sihag, Bhaskar Borah, Rocky Pebam, Harpreet Kaur, Kalpana Baruah, Kanwar Narain, Sarala K. Subbarao, Dibya Ranjan Bhattacharyya, Biswajyoti Borkakoty, Ipsita Pal Bhowmick

**Affiliations:** 1 Regional Medical Research Center- Northeast Region (RMRC-NE)-ICMR, Dibrugarh, 786001 India; 2 Regional Office of Health and Family Welfare, Kolkata, 700106 India; 3 Indian Council of Medical Research (ICMR), Ramalingaswami Bhavan, Delhi, 110029 India; 4 National Center for Vector Borne Disease Control, DGHS, 110054 MOHFWNew Delhi, India; 5 Formerly Regional Medical Research Center- Northeast Region (RMRC-NE)-ICMR, Dibrugarh, 786001 India; 6 Formerly National Institute of Malaria Research, ICMR, 110077 Delhi, India; 7 Centre for Post Graduate & Research Studies, Periyar University, Dharmapuri, 635205 India; 8 National Health Mission (NHM), Biswanath, Assam India; 9 Department of Space, North East Space Application Centre (NESAC), Government of India, Umiam, 793103 India; 10 Department of Health Research, Model Rural Health Research Unit (MRHRU), Tripura, GovtAgartala India

Correction to: *Scientific Reports* 10.1038/s41598-025-96146-9, published online 24 April 2025

The original version of this Article contained an error in the Abstract.

“The *Aedes* (*Stegomyia*) *albopictus* (Skuse, 1985) (Diptera: Culicidae) is one of the major vectors for Dengue and Chikungunya.”

now reads:

“The *Aedes* (*Stegomyia*) *albopictus* (Skuse, 1895) (Diptera: Culicidae) is one of the major vectors for Dengue and Chikungunya.”

In addition, in the Introduction section,

“Among these, *Aedes* (*Stegomyia*) *aegypti* (Linnaeus, 1762) and *Aedes* (*Stegomyia*) *albopictus* (Skuse, 1985) are particularly well-known for being primary vectors of Dengue, Chikungunya, and Zika virus.”

now reads:

“Among these, *Aedes* (*Stegomyia*) *aegypti* (Linnaeus, 1762) and *Aedes* (*Stegomyia*) *albopictus* (Skuse, 1895) are particularly well-known for being primary vectors of Dengue, Chikungunya, and Zika virus.”

“In India, *Ae. albopictus*, *Ae. novalbopictus*, *Ae. patriciae*, *Ae. pseudalbopictus*, *Ae. subalbopictus*, *Ae. unilineatus*, *Ae. krombeini*, *Ae. malayensis*, and *Ae. scutellaris* have been reported under Scutellaris Group^6^.”

now reads:

“In India, Ae. albopictus, Ae. novalbopictus, Ae. patriciae, Ae. pseudoalbopictus, Ae. subalbopictus, Ae. unilineatus, Ae. krombeini, Ae. malayensis, and Ae. scutellaris have been reported under Scutellaris Group^6^.”

Additionally, in the Discussion section,

“Phylogenetic analyses (Figs. 2 and 3) conclusively indicate the presence of this species which is morphologically similar to *Ae. albopictus* but genetically different from *Ae. albopictus*, *Ae. subalbopictus*, *Ae. pseudoalbopictus*, *Ae. flavopictus*, *Ae. aegypti*, *Ae. vittatus*, while resembling *Ae. albopictus* cryptic species from China and Vietnam.”

now reads:

“Phylogenetic analyses (Figs. 2 and 3) conclusively indicate the presence of this species which is morphologically similar to *Ae. albopictus* but genetically different from *Ae. albopictus*, *Ae. subalbopictus*, *Ae. flavopictus*, *Ae. aegypti*, *Ae. vittatus*, while resembling *Ae. albopictus* cryptic species from China and Vietnam.”

The original Article has been corrected.

