# Supplementary material for: Detection of a sympatric cryptic species mimicking Aedes albopictus (Diptera: Culicidae) in dengue and Chikungunya endemic forest villages of Tripura, India, posing a daunting challenge for vector research
Source: Sci Rep. 2025 Apr 24;15:14237. doi: 10.1038/s41598-025-96146-9 (PMC12022023; doi:10.1038/s41598-025-96146-9)
Supplement: Supplementary file 1 — Supplementary Material 1 [file 41598_2025_96146_MOESM1_ESM.docx]

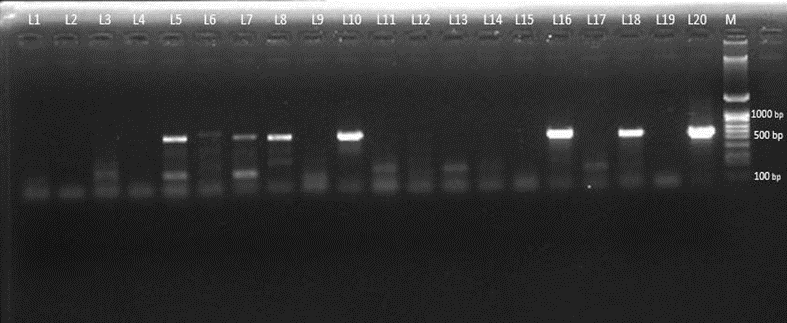


**Supplementary Figure 1:** Representative gel image of *Wolbachia* detection. L1-L4, L9, L11-L14 represent the absence of *Wolbachia* in the *Aedes nr. albopictus* specimens. L5-L8, L10, L16, L18 represent the presence of *Wolbachia* in *Aedes albopictus* via amplification of *wsp* gene while L15 and L17 did not show any amplification in *Aedes albopictus*. L19 and L20 represent negative and positive control respectively.
